# Structural Transition of the Nucleosome during Transcription Elongation

**DOI:** 10.3390/cells12101388

**Published:** 2023-05-14

**Authors:** Tomoya Kujirai, Haruhiko Ehara, Shun-ichi Sekine, Hitoshi Kurumizaka

**Affiliations:** 1 Laboratory of Chromatin Structure and Function, Institute for Quantitative Biosciences, The University of Tokyo, 1-1-1 Yayoi, Bunkyo-ku, Tokyo 113-0032, Japan; kujirai@iqb.u-tokyo.ac.jp; 2 Laboratory for Transcription Structural Biology, RIKEN Center for Biosystems Dynamics Research, 1-7-22 Suehiro-cho, Tsurumi-ku, Yokohama 230-0045, Japan; haruhiko.ehara@riken.jp (H.E.); shunichi.sekine@riken.jp (S.-i.S.)

**Keywords:** nucleosome, chromatin, transcription, RNA polymerase II, histone chaperone, FACT, template DNA looping

## Abstract

In eukaryotes, genomic DNA is tightly wrapped in chromatin. The nucleosome is a basic unit of chromatin, but acts as a barrier to transcription. To overcome this impediment, the RNA polymerase II elongation complex disassembles the nucleosome during transcription elongation. After the RNA polymerase II passage, the nucleosome is rebuilt by transcription-coupled nucleosome reassembly. Nucleosome disassembly–reassembly processes play a central role in preserving epigenetic information, thus ensuring transcriptional fidelity. The histone chaperone FACT performs key functions in nucleosome disassembly, maintenance, and reassembly during transcription in chromatin. Recent structural studies of transcribing RNA polymerase II complexed with nucleosomes have provided structural insights into transcription elongation on chromatin. Here, we review the structural transitions of the nucleosome during transcription.

## 1. Introduction

In eukaryotes, genomic DNA encoding genetic information is stored in the nucleus, where it is folded into chromatin with histone proteins. The basic unit of chromatin is the nucleosome, composed of four core histones, H2A, H2B, H3, and H4, and about 150 base pairs of DNA [1,2,3]. Chromatin is a macromolecular complex, in which nucleosomes are connected by linker DNAs with a beads-on-a-string appearance. The DNA is tightly bound to the histones in the nucleosome, and thus the nucleosome is essentially inhibitory to genomic transcription, replication, recombination, and repair [4].

During transcription processes, RNA polymerase II (RNAPII) must overcome multiple nucleosome barriers. To do so, the RNAPII elongation complex (EC) transcribes genes with concomitant disassembly of the nucleosome. Afterwards, the chromatin structure is mostly restored in the transcribed regions [5,6]. This nucleosome maintenance is essential for cell survival, and disordered chromatin structures negatively impact transcription fidelity. For example, chromatin structure disruption leads to cryptic transcription, in which aberrant RNAs are synthesized from non-promoter regions [7]. Chromatin abnormalities also induce a loss of epigenetic information such as histone modifications, which play regulatory roles in transcription, splicing, DNA methylation, and RNA methylation [6,7,8,9].

During transcription elongation on chromatin, the histone chaperone FACT facilitates nucleosome disassembly, maintenance, and reassembly [10,11,12]. As an alternative mechanism, template DNA looping, in which the transcribed DNA folds back into the nucleosome, may also be an intermediate in the nucleosome transfer process [5,13,14]. Recent cryo-EM analyses have provided snapshot structures of the transcribing RNAPII-nucleosome complexes, thus clarifying the mechanisms of chromatin transcription and epigenetic regulation in three-dimensional structures [15,16] and revealing the various nucleosome-binding modes of FACT during transcription elongation. In this review, we focus on the dynamic structural transitions in nucleosome disassembly and reassembly processes during transcription elongation, based on recently reported structures.

## 2. The Nucleosome Structure

**Figure 1:**
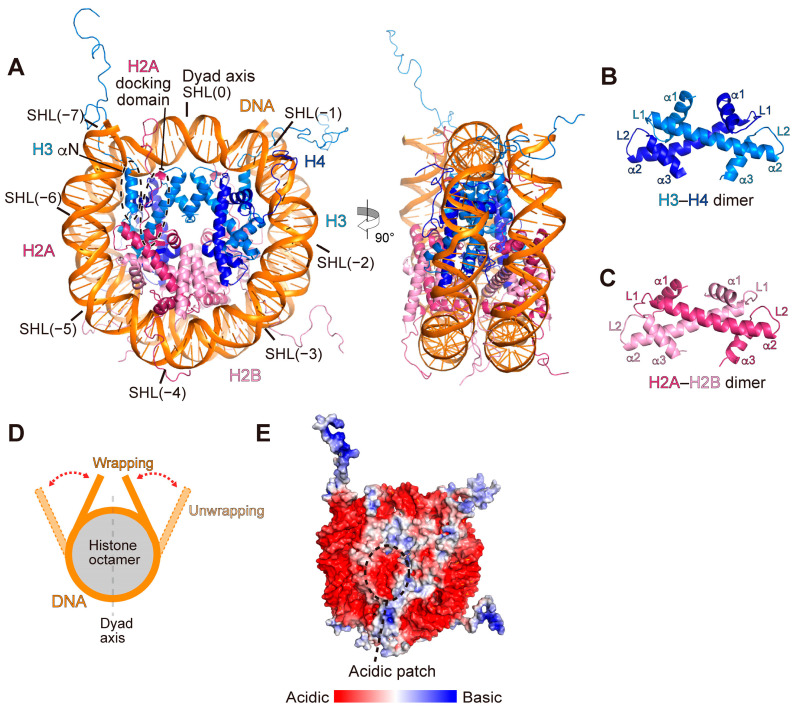
Nucleosome structure. (**A**) Crystal structure of the nucleosome (PDB: 1KX5). The superhelical locations (SHLs) on the nucleosome are indicated. The αN helix and the H2A-docking domain are highlighted. (**B**,**C**) The histone-fold structures of (**B**) the H3–H4 dimer and (**C**) the H2A–H2B dimer, extracted from the nucleosome structure. (**D**) Nucleosome breathing. The entry/exit sites of the nucleosomal DNA transiently wrap around and unwrap from the histone octamer, as indicated by the red dotted arrows. (**E**) Surface electrostatic potential of the nucleosome.

The nucleosome is a disk-like structure, consisting of a histone octamer with about 150 base pairs of DNA wrapped around it [4,17,18] (Figure 1A).The histone octamer contains two molecules of the four histone proteins H2A, H2B, H3, and H4, which form heterodimers in specific combinations: H3–H4 and H2A–H2B (Figure 1B,C). Histone heterodimers are formed through structurally conserved histone-fold motifs. In addition, the flexible tails are preserved in the N-terminal regions of all four histones, and a long C-terminal tail extends from H2A.

In the nucleosome, two H3–H4 dimers associate and form a four-helix bundle between two H3 molecules (H3–H4 tetramer formation) (Figure 1A). The H2A–H2B dimer associates with the H3–H4 tetramer by forming another four-helix bundle between H2B and H4. In addition, the H2A C-terminal region contains a docking domain, which interacts with H3–H4, and particularly with the H3 αN helix (Figure 1A). The docking domain of H2A includes the regulating-octamer-folding (ROF) region, and its loss leads to the dissociation of the H3–H4 tetramer and the H2A–H2B dimer [19,20]. The N-terminal tails are extended from the nucleosome core and exposed to the solvent.

In the nucleosome, the histone octamer directly binds to the DNA backbone using basic lysine and arginine residues, with about 10 base-pair periodicity. The nucleosome structure has a pseudo-twofold symmetry axis called the dyad axis. The positions on the nucleosomal DNA are defined as superhelical locations (SHLs). The center of the nucleosomal DNA, where the minor groove faces outwards and intersects the dyad axis of the nucleosome, is designated SHL(0). Based on the position of SHL(0), the positions of every minor groove facing outwards are defined as SHLs (−7), (−6), (−5), (−4), (−3), (−2), (−1), (+1), (+2), (+3), (+4), (+5), (+6), and (+7). In the nucleosome, the H2A–H2B dimer interacts with DNA at SHLs (±5), (±4), and (±3), and the H3–H4 tetramer interacts with DNA at SHLs (±7), (±6), (±2), (±1), and (+0) [4,18] (Figure 1A). The entry/exit sites of the nucleosomal DNA regions from SHL(±7) to SHL(±5) are more flexible than the other nucleosomal DNA regions, as revealed by biophysical and structural analyses [21,22] (Figure 1D). This characteristic has been termed “nucleosome breathing” [23]. Nucleosome breathing may facilitate the interactions of DNA binding proteins with the entry/exit regions of nucleosomal DNA. Molecular dynamics simulation analyses suggested that nucleosome breathing is directly regulated by the H2A C-terminal and H3 N-terminal tails [24,25]. Consistently, deletions of the histone tails, particularly the H3 tail, as well as acetylation of the H3 tail, increase nucleosome breathing in vitro, and proteolytic removal of histone tails induces partial unwrapping of the nucleosomal DNA [26,27]. A nucleosome lacking histone tails can be stabilized by the histone chaperone FACT [26].

The surface of the nucleosome has a cluster of acidic amino acid residues, termed the acidic patch (Figure 1E). This acidic patch was first shown to bind the viral protein LANA from Kaposi’s herpes sarcoma virus, and many nucleosome-binding factors were subsequently identified as acidic patch binding proteins [28]. A pocket within this patch captures an arginine side chain of nucleosome-binding proteins (referred to as the “arginine anchor”).

## 3. DNA Peeling during Nucleosome Transcription

In gene expression, the DNA sequences of gene coding regions are transcribed into messenger RNA by RNAPII. The genome-wide localization of RNAPII has been analyzed at single base-pair resolution by sequencing the 3’ ends of transcripts with next-generation sequencers [29,30]. Nucleosomes act as barriers to RNAPII in cells, and RNAPII is paused near the nucleosomal entry region and the center [30,31]. The RNAPII-pausing positions in cells are generally consistent with those observed in in vitro experiments [30,32].

**Figure 2:**
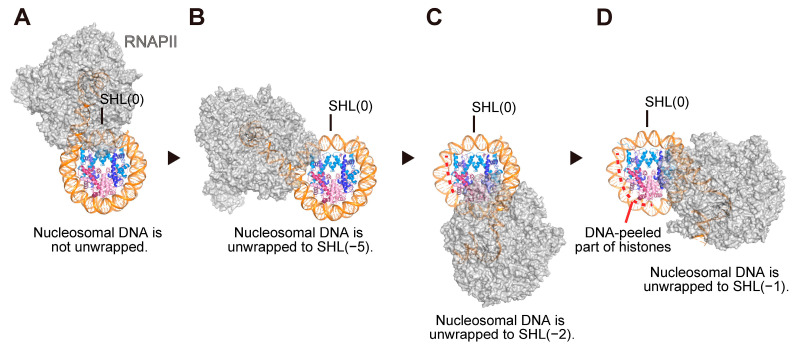
Structures of nucleosomes during transcription by RNAPII. The leading edge of the RNAPII proceeds to SHL(−6) (**A**), SHL(−5) (**B**), SHL(−2) (**C**), and SHL(−1) (**D**). The exposed surfaces of the histones are indicated. PDB IDs: 6A5O (**A**), 6A5P (**B**), 6A5R (**C**), and 6A5T (**D**).

Recent innovations in cryo-EM technology have enabled high-resolution structural analyses of various chromatin complexes [33,34]. In contrast to previous low-resolution structures of RNA polymerase-nucleosome complexes [35], detailed analyses of RNAPII-nucleosome complexes have been reported [36,37,38,39,40,41,42]. For structural analyses, a template nucleosome with a linker DNA containing an RNAPII binding site, where RNAPII starts RNA elongation, was employed to reproduce nucleosomal transcription in vitro. Using this transcription system, snapshot structures of the transcribing RNAPII-nucleosome complexes, in which RNAPII was paused at SHLs (−6), (−5), (−2), and (−1) in the nucleosome, were determined through cryo-EM single-particle analysis [37] (Figure 2A–D). These structures revealed that the nucleosomal DNA is gradually peeled by RNAPII during transcription elongation, and the nucleosomal histones are maintained as an octamer containing two of each histone dimer, H2A–H2B and H3–H4. It should be noted that the structure of a backtracked RNAPII elongation complex with the hexasome, in which the promoter-proximal H2A–H2B dimer is dissociated from the complete nucleosome, has also been reported [40]. The dissociation of the H2A–H2B dimer may be regulated by various factors, such as RNAPII stalling/backtracking, histone chaperones, salt concentration, etc.

RNAPII pauses at major histone–DNA contact sites, suggesting that histone–DNA interactions play regulatory roles in transcription elongation by RNAPII [43,44]. Histone acetylation may weaken the interactions at histone–DNA contacts by neutralizing basic residues [45,46]. In the nucleosome, acetylations of lysine residues at positions 56, 64, and 122 of H3 are reportedly involved in transcription activation in vivo [47,48,49]. Consistent with these observations, RNAPII efficiently transcribed acetylated nucleosomes in vitro [50,51]. Moreover, the histone variant H2A.Z and ubiquitinated H2B affected transcription efficiency [52]. Sin mutations that destabilize the nucleosome structure promoted efficient transcription on the nucleosome [53,54,55]. A histone mutation located in the entry/exit site of the nucleosome impaired transcription fidelity, as revealed by transcription termination defects and pervasive transcription [56]. In addition, histone tails facilitate RNAPII pausing around the nucleosomal entry region [57,58].

Histones are eventually dissociated from DNA when RNAPII passes through the central position of the nucleosome during transcription elongation [30,42]. However, nucleosomes containing epigenetic information, such as post-translational modifications and histone variants, are maintained even in actively transcribed regions in vivo, suggesting the existence of a transcription-coupled nucleosome maintenance mechanism [6,14,15,29,59]. During RNAPII passage through the nucleosome, the DNA-binding surface of the nucleosomal H2A–H2B dimer is exposed and can bind a variety of factors, including the histone chaperone FACT, cis/trans DNA fragments, and transcription elongation factor SPT5, which are involved in nucleosome maintenance during transcription elongation [37,38,39,42,60,61,62].

## 4. Histone Chaperone FACT

**Figure 3:**
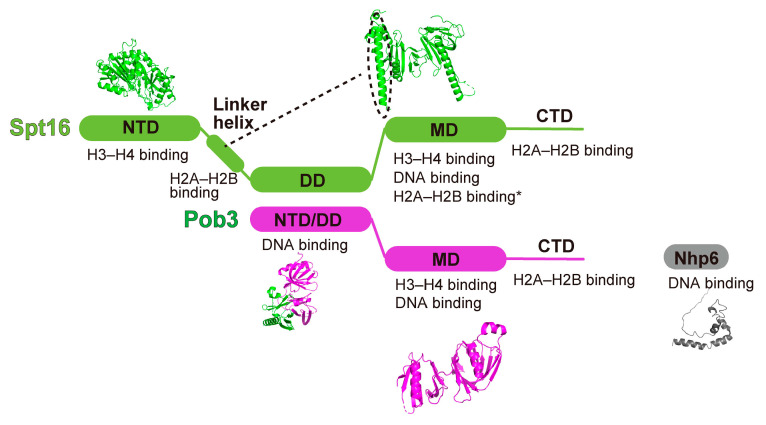
FACT structure. The primary domains with the three-dimensional structures of yeast FACT are shown. The interactions between FACT domains and histone complexes are described. *: Interaction detected in the Spt16MD—H2A–H2B dimer fusion protein. NTD: N-terminal domain, DD: dimerization domain, MD: middle domain, CTD: C-terminal domain. PDB IDs: 5UMT (Spt16 NTD), 7XSX (Spt16 MD with the linker helix), 4KHB (DD), 2GCL (Pob3 MD), 1CG7 (Nhp6).

The histone chaperone FACT was identified in 1998 as a protein complex that FAcilitates Chromatin Transcription by RNAPII [63]. FACT is a heterodimer of the Spt16 and Ssrp1 (Pob3 in yeast) proteins, and is conserved from yeasts to humans [64,65] (Figure 3). Spt16 has an N-terminal aminopeptidase domain (NTD), a dimer formation domain (DD) with Ssrp1/Pob3, a middle domain (MD) consisting of tandem pleckstrin-homology (PH1 and 2) domains, and a C-terminal domain (CTD) [66,67,68,69,70]. In addition, a linker helix, located between the NTD and DD in the primary structure of Spt16, is integrated within the Spt16 MD [42]. Pob3 consists of a dimer-formation domain (DD) at the N-terminal end, a middle domain (MD) consisting of tandem pleckstrin-homology (PH1 and 2) domains, and a C-terminal domain (CTD) [69,71,72]. Spt16 and Pob3 were originally found as a suppressor of the Ty6 retrotransposon and a binding factor for the DNA polymerase alpha subunit DNA pol1 (polymerase one binding factor 3; Spt16 is Pob2), respectively [10]. Ssrp1, a metazoan homolog of Pob3, additionally has an HMGB-like DNA binding domain at its C-terminal region, compared to Pob3 [73]. In yeast, an HMGB-like protein, Nhp6 (non-histone protein 6), exists as a separate protein component that associates with FACT [74,75], and is considered to be the counterpart of the HMGB domain in Ssrp1 [76].

Many FACT domains have been shown to bind DNA and histone complexes [42,60,61,66,69,70,72,77,78]. The interactions between histones and FACT domains, based on biochemical and structural analyses, are summarized in Figure 3. The aminopeptidase domain of Spt16 is evolutionally conserved; however, no peptidase activity has been detected because the active center residues of the peptidase domain are altered [66]. Consistent with the inactive peptidase activity of Spt16, the peptidase domain reportedly does not bind to the N-terminal tails of histones [67].

## 5. Functions of FACT

In the genome, FACT is localized in the gene body regions along with RNA polymerase [79,80,81,82,83,84,85]. FACT performs complicated functions: promoting nucleosome disassembly, maintaining sub-nucleosome structure, and facilitating nucleosome reassembly [10,11,12]. An early report suggested that FACT functions to destabilize and change the nucleosome structure, and promote RNAPII elongation in chromatin [86]. Indeed, FACT reportedly activated nucleosomal DNA transcription in vitro [39,87,88]. FACT altered nucleosome structure and maintained its state with increased DNA accessibility [89,90]. These reports indicate that FACT promotes the disassembly of the nucleosome while preserving the partially unwrapped sub-nucleosome structure.

FACT plays a role in nucleosome reassembly after RNA polymerase passage through the nucleosome [10,11,59,83,91,92]. In vitro, FACT assembled a nucleosome and promoted nucleosome retention during transcription [60,87,88,93]. FACT prevented cryptic transcription, which occurs when nucleosomes are not properly restored after transcription [10,79,94,95,96,97]. Transcription-dependent loss of old histone proteins and incorporation of new histone proteins were observed upon acute inactivation of FACT, suggesting that FACT transfers histones dissociated from the genomic DNA by RNAPII passage to the region behind RNAPII [91]. This FACT-mediated nucleosome reassembly during transcription by RNAPII contributes to the maintenance of epigenetic information, as histone H3 K4 methylation, K36 methylation, and the H2A.Z variant are retained by FACT during transcription [98,99,100].

FACT is highly abundant in yeast cells, which have ~42,000 molecules of FACT and over ~75,000 nucleosomes per cell, suggesting that nearly half of the nucleosomes may interact with FACT [101].

## 6. FACT–Nucleosome Structures

**Figure 4:**
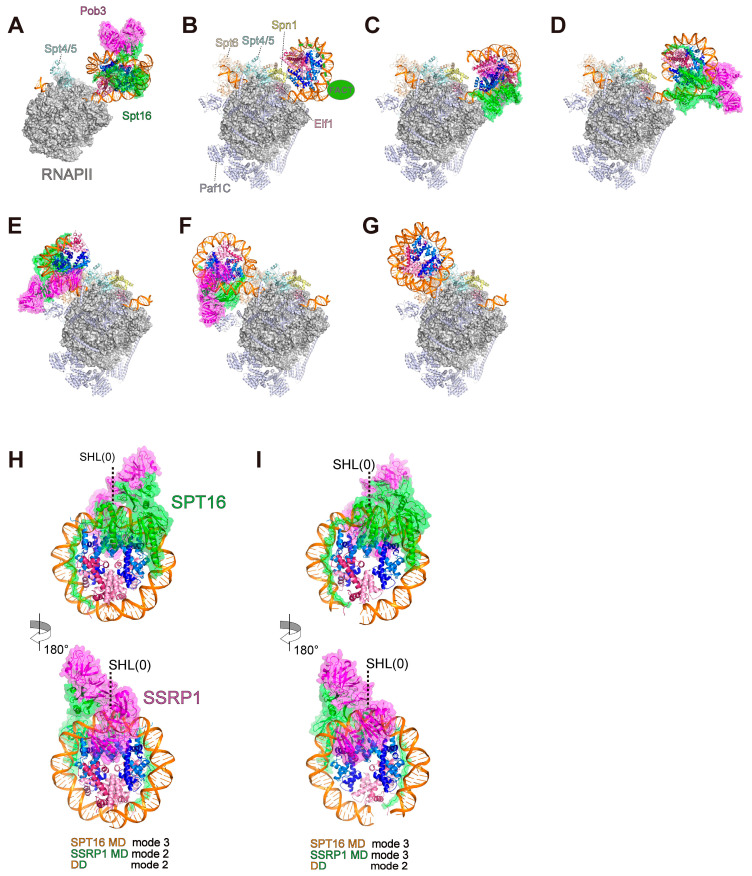
Structures of FACT–nucleosome complexes. (**A**–**D**) Structures of the RNAPII–FACT–downstream nucleosome complexes, in which RNAPII proceeded by 17 bases (**A**), 42 bases (**B**), 49 bases (**C**), and 49 bases (**D**) from the nucleosomal entry. Note that the nucleosome–FACT complex in panel D had been transferred downstream. (**E**–**G**) Structures of RNAPII–FACT–nucleosome complexes, in which RNAPII proceeded by 58 bases (**E**), 58 bases (**F**), and 115 bases (**G**) from the nucleosomal entry. (**H**–**I**) Structures of reconstituted FACT complexes with octasome (**H**) and hexasome (**I**). PDB IDs: 7NKY (**A**), 7XSE (**B**), 7XSX (**C**), 7XT7 (**D**), 7XTI (**E**), 7XTD (**F**), 7XSZ (**G**), 6UPL (**H**), and 6UPK (**I**).

The establishment of reconstitution methods for both FACT–nucleosome complexes and the intermediate complexes of nucleosomal DNA transcription by RNAPII allowed for the visualization of three-dimensional structures of the FACT–nucleosome complexes [39,42,60,61,78,88]. Farnung et al. reported a cryo-EM structure in the nucleosome–FACT–RNAPII EC containing Spt4/5, in which the EC proceeded 17 bases from the nucleosomal entry and the leading edge of the RNAPII paused around SHL(−4) in the nucleosome [39] (Figure 4A). Ehara et al. reported six structures of EC–nucleosome–FACT complexes, EC42, EC49, EC49B, EC58^hex^, EC58^oct^, and EC115, in which RNAPII proceeded by 42, 49, 58, and 115 bases from the nucleosomal entry [42] (Figure 4B–G). The EC contains transcription elongation factors Paf1C, Spt6, Spn1, Elf1, and Spt4/5. The leading edge of the EC is located around SHL(−1) for EC42 and EC49B, SHL(0) for EC49, SHL(+1) for EC58^hex^ and EC58^oct^, and SHL(+6) for EC115 in the nucleosomal DNA. In the SHL(−1) and SHL(0) complexes, the FACT–nucleosome complex is downstream of the EC, while in the SHL(+1) and SHL(+6) complexes, the nucleosome is transferred upstream of the EC. Liu et al. reconstituted the complexes formed during nucleosome assembly by FACT by mixing DNA, H2A–H2B dimer, H3–H4 tetramer, and FACT, and determined two structures in FACT–nucleosome complexes [60] (Figure 4H,I). One structure is a complex with an octasome containing two H2A–H2B and H3–H4 dimers, and the other is a complex with a hexasome, in which an H2A–H2B dimer is missing.

In these structures, Spt16 MD and Ssrp1/Pob3 MD bind to opposite surfaces of the nucleosome disk, and DD sits near the nucleosomal DNA SHL(0). Interestingly, this series of structures revealed multiple modes by which FACT binds to the nucleosome. Here, we have classified the modes of FACT binding to the nucleosome (Table 1, Table 2, Table 3 and Table 4). The binding modes of Spt16 MD, Pob3 MD, and DD are described below.

**Figure 5:**
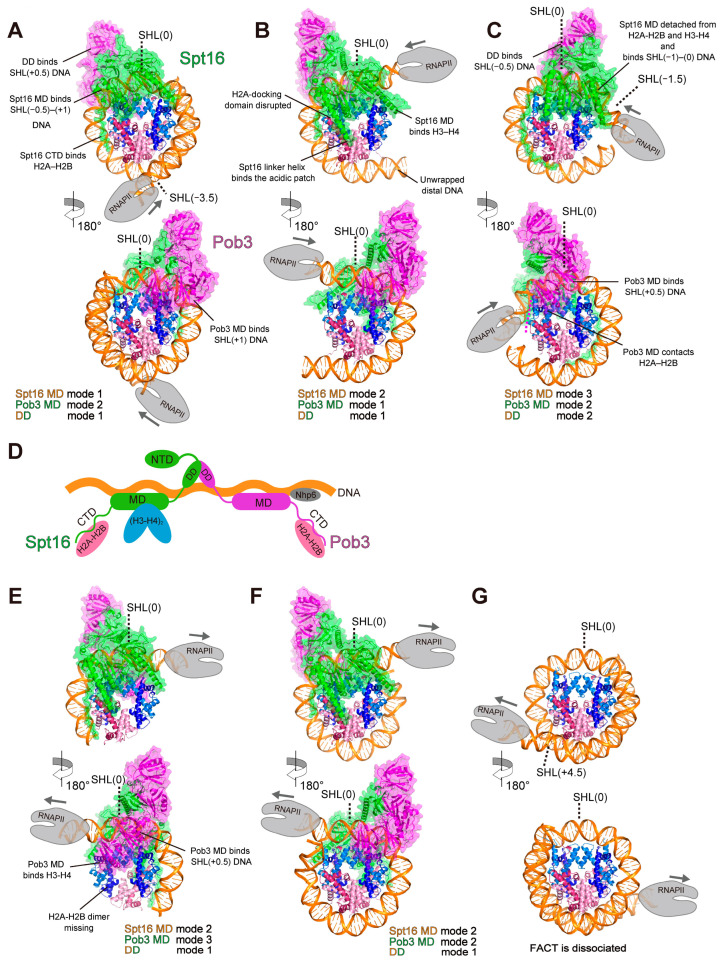
Structural transition of the nucleosome during transcription in the presence of FACT. (**A**–**C**) Downstream nucleosome structures in which RNAPII proceeded 17 bases (**A**), 49 bases (**B**), and 49 bases (**C**) from the nucleosomal entry. Note that the nucleosome–FACT complex in panel (**C**) had been transferred downstream after RNAPII reached the area around SHL(0). (**D**) Proposed model of the extended conformation of the FACT–nucleosome complex. (**E**–**F**) Upstream nucleosome structures in which RNAPII proceeded by 58 bases (**D**), 58 bases (**E**), and 115 bases (**F**) from the nucleosomal entry. Features of each mode are shown. These nucleosome structures are extracted from PDB IDs 7NKY (**A**), 7XSX (**B**), 7XT7 (**C**), 7XTI (**E**), 7XTD (**F**), and 7XSZ (**G**).

Three binding modes of Spt16 MD have been found (Figure 5A–C upper panels; Table 1). In mode 1, the Spt16 MD binds to the nucleosomal DNA region from SHL(−0.5) to SHL(+1) without obvious interactions with the H3–H4 tetramer, and the Spt16 CTD binds to the exposed promoter-proximal H2A–H2B in the nucleosome (Figure 5A upper panel). In mode 2, the Spt16 MD directly binds to the histones (Figure 5B upper panel). The Spt16 MD interacts with the DNA-peeled surface of H3–H4 around SHL(−1) of the nucleosome. The H2A-docking domain and the H3 αN helix are displaced by the Spt16 MD, indicating that FACT disrupts the interaction between the H2A–H2B and H3–H4 dimers in the nucleosome. The interaction between Spt16 MD and the H3–H4 tetramer in the nucleosome is similar to that in the crystal structure of the H3–H4 tetramer–Spt16 MD complex [78]. The arginine residue located at the edge of the linker helix could interact with the acidic patch of the proximal H2A–H2B in the nucleosome. The Spt16 CTD binds to the exposed H2A–H2B in the nucleosome. Thus, Spt16 binds to the H3–H4 tetramer and the proximal H2A–H2B dimer in the nucleosome, suggesting that it can directly contact a histone hexamer. In mode 3, the Spt16 MD is detached from the histones and mainly binds to the nucleosomal DNA around SHL(−1)-SHL(0) (Figure 5C upper panel), while the Spt16 CTD remains bound to the DNA-peeled surface of H2A–H2B.

Three binding modes of Ssrp1/Pob3 MD have been found (Figure 5A-C lower panels; Table 2). In mode 1, Ssrp1/Pob3 MD binds to the nucleosomal DNA around SHL(+1), apparently without direct interactions with the nucleosomal histones (Figure 5A lower panel). In mode 2, Ssrp1/Pob3 MD binds to the nucleosomal DNA around SHL(+0.5) and contacts the H2A–H2B dimer (Figure 5B lower panel). In mode 3, Ssrp1/Pob3 MD contacts the H3 four-helix bundle and the H3 α2 helix, which form the H2A–H2B dimer binding site of the H3–H4 tetramer. Ssrp1/Pob3 MD also binds the nucleosomal DNA around SHL(+0.5) (Figure 5C lower panel).

Two binding modes of DD have been reported (Figure 5A–C; Table 3). In mode 1, DD binds to the nucleosomal DNA around SHL(+0.5) (Figure 5A,B). In mode 2, DD binds to the nucleosomal DNA around SHL(−0.5) (Figure 5C).

The classifications of the binding modes on the reported FACT–nucleosome structures are summarized in Table 4, which shows five combinations of binding modes. The modes of Spt16 MD, DD, and Ssrp1/Pob3 MD binding are constrained to each other because each domain is connected by a peptide linker (Figure 3), suggesting the existence of possible combinations. The constraint between Spt16 MD and Ssrp1/Pob3 MD may be involved in regulation of the disassembly, maintenance, and reassembly of the nucleosome.

## 7. Structural Transition of FACT Binding to the Nucleosome during Transcription Elongation

During nucleosome transcription by RNAPII, Spt16 and Pob3 undergo major changes in their binding modes. Here, we summarize the transition of FACT binding to the nucleosome during transcription elongation, based on the reported structures of FACT–nucleosome–RNAPII complexes.

When the EC invades the nucleosome and peels the nucleosomal DNA around SHL(−4), Spt16 first binds in mode 1 through Spt16 CTD binding to the exposed promoter-proximal H2A–H2B [39] (Figure 5A, upper panel). This Spt16 binding is in good agreement with biochemical and genome-wide analyses showing that FACT binds to a deformed nucleosome, and not to an intact nucleosome [78,84,98]. When the EC reaches SHL(−1), FACT is not tightly bound to the nucleosome at this stage, while the Spt16 CTD may remain bound to the exposed H2A–H2B (Figure 4B). When the EC reaches SHL(0), Spt16 shifts to mode 2 through Spt16 MD binding to the exposed H3–H4 around SHL(−1) [42] (Figure 5B upper panel). In mode 2, Spt16 binds the proximal H2A–H2B dimer on the nucleosome through Spt16 linker helix binding to the acidic patch. This observation is consistent with reports that FACT prevents the loss of the proximal H2A–H2B dimer in vivo and in vitro, and mutations in the acidic patch lead to decreased nucleosome occupancy in transcribed regions [87,102,103,104]. In addition, the promoter-distal side of the nucleosomal DNA around SHL(+7)–(+5) is unwrapped, due to steric hindrance with RNAPII and the promoter-proximal nucleosomal DNA. In this position, ~70% of the histone–DNA contact is lost, implying that the FACT–histone complex may be easily dissociated from the DNA fragment. It should be noted that since the Spt16 MD reportedly promotes the H2A–H2B dimer displacement, the proximal H2A–H2B dimer may be dissociated from the histone octamer and tethered by the Spt16 CTD in this position [78,93]. When the FACT–histone complex jumps downstream, which accompanies DNA rewrapping around SHL(−1), Spt16 shifts to mode 3 through Spt16 MD dissociation from the nucleosomal histones (Figure 5C upper panel). It is possible that mode 3 reverts to mode 2 when the DNA is again peeled by the EC [42,60]. The distal nucleosomal DNA end is still unwrapped, and the Pob3 CTD is bound to the exposed surface of the distal H2A–H2B dimer. These structural transitions demonstrate that FACT changes its binding modes depending on the DNA wrapping situation of the nucleosome.

After the EC has passed through the nucleosomal DNA around SHL(0), the nucleosome is dissociated from the downstream DNA and transferred [30,42]. As an intermediate structure of the FACT–nucleosome complex during transfer, the existence of an Spt16-histone hexamer complex with an H2A–H2B dimer tethered by Pob3 has been proposed [42]. Additionally, based on two-dimensional averaged images in the EM analysis of the FACT–nucleosome complex in the presence of Nhp6, an extended conformation model of the FACT–nucleosome complex has been suggested. In this model, the Spt16 CTD, Spt16 MD, and Pob3 CTD tether an H2A–H2B dimer, an H3–H4 tetramer, and another H2A–H2B dimer, respectively [105] (Figure 5D). This extended FACT–histone complex could also be the intermediate complex during nucleosome transfer. The FACT–histone complex may adopt various conformations during the transfer from downstream to upstream of the EC during transcription elongation. Although whether the FACT–histone complex is transferred in cis or trans is unknown in vitro, the complex in vivo must be transferred in cis so that FACT can maintain the epigenetic information in the genome.

In the initial phase of nucleosome reassembly after nucleosome transfer, FACT–histone hexasome and octasome complexes have been observed on the upstream surface of the EC, which is composed of transcription elongation factors. This hexasome formation is consistent with reports that the H2A–H2B dimer is dissociated from chromatin in a transcription-dependent manner in vivo [106,107,108]. Spt16 binds to the histone octasome or hexasome in mode 2 (Figure 5E,F upper panels). In contrast, Pob3 differentially binds to the histone octasome and hexasome in modes 2 and 3, respectively (Figure 5E,F lower panels; Table 2), suggesting that Pob3 may change its binding mode depending on the presence of the H2A–H2B dimer [42,60]. Since the Pob3 CTD binds the H2A–H2B dimer, Pob3 mode 3 may promote the deposition of the H2A–H2B dimer on the hexasome [42,70,93,105]. After the initial reassembly phase, the nucleosomal DNA is rewrapped and then RNAPII progression continues (Figure 5G). In this stage, FACT dissociates from the nucleosome, probably because its binding site is blocked by the rewrapped DNA.

These structural transitions show that FACT changes its binding modes to facilitate nucleosome disassembly, maintenance, and reassembly in response to the RNAPII position on the nucleosome. When RNAPII peels the nucleosomal DNA, FACT promotes nucleosome disassembly and maintains the sub-nucleosome structure. After RNAPII passes through the nucleosome, FACT facilitates the reassembly of the sub-nucleosome structure and matures it into a complete nucleosome.

## 8. Structural Diversity of Nucleosomes in Complexes with FACT

**Figure 6:**
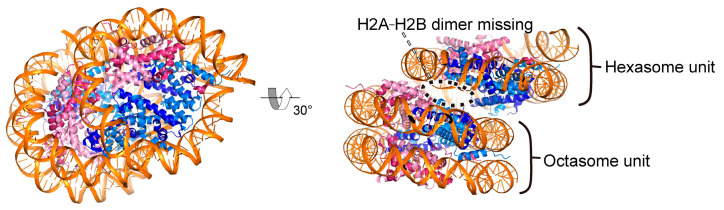
Structure of the overlapping di-nucleosome (PDB ID: 5GSE). The hexasome and octasome units are indicated.

FACT may also bind an unusual di-nucleosome. DNA fragments of inter-nucleosomal size (~150–300 base pairs) were detected around the +1 nucleosome in ChIP-seq analysis of Spt16 with micrococcal nuclease (MNase) digestion, in addition to those of sub-nucleosomal size (~80 base pairs) [84,85]. Although it is possible that these inter-nucleosomal-sized DNA fragments originated from complexes containing other factors besides FACT and histones, such DNA fragments were observed in an MNase treatment assay during poly-nucleosome remodeling reactions by switch-sucrose nonfermentable (SWI/SNF) remodeling factors in vitro [109]. This unusual di-nucleosome is designated as an “overlapping di-nucleosome”, in which two nucleosomes are tightly associated with each other [110]. The crystal structure of the overlapping di-nucleosome revealed that it consists of a histone octasome and hexasome with 250 base pairs of DNA (Figure 6; [111]). In the structure, the side of the hexasome lacking H2A–H2B contacts the octasome. A genome-wide localization analysis of 250 base pair DNA fragments from MNase-digested chromatin revealed their enrichment near the +1 nucleosome, which is located just downstream of the promoter region. This localization is in good agreement with that of the inter-nucleosomal-sized fragments detected by MNase-ChIP-seq analysis of Spt16 [84,85,111]. These reports implied that a FACT-overlapping di-nucleosome complex may be formed around the +1 nucleosome region, possibly as a result of a collision between the +1 and +2 nucleosomes induced by RNAPII and FACT. Since the overlapping di-nucleosome, like the closed-packed di-nucleosome, may inhibit transcription, it could be resolved by chromatin remodeling factors such as Chd1 and Isw1 [112].

## 9. Template DNA Looping-Mediated Nucleosome Retention

During transcription elongation by RNAPII, the DNA-peeled H2A–H2B region in the nucleosome serves as not only a FACT-binding site but also a DNA-binding site for a cis/trans DNA fragment [37,41]. This suggests that the DNA bound to the histone complex may be an intermediate structure for histone transfer in cis or in trans. Specifically, the template DNA forms a loop through the RNAP, wherein the transcribed DNA behind the RNAP is folded back to the DNA-peeled surface of the histone complex. This template DNA looping model has also been proposed as a mechanism of nucleosome retention [5,13,14,15,113,114,115]. In this model, after the histone complex is dissociated from the template DNA fragment, the histone octamer is transferred upstream of the RNAP through the loop. The transferred nucleosome could be a hexasome or an octasome, probably depending on the RNAPII elongation rate [115,116]. The hexasome formation is consistent with in vivo observations that the amount of the H2A–H2B dimer is reduced in actively transcribed regions [106,107,108]. Template DNA looping-mediated nucleosome retention accompanies nucleosome repositioning, depending on the loop size. A small DNA loop that does not affect nucleosome repositioning (called the zero-loop) has also been proposed [116,117]. To date, eukaryotic RNAPII, RNAPIII, bacteriophage SP6 RNAP, and *Escherichia coli* (*E. coli*) RNAPs have been reported to form template DNA loops in vitro [41,113,114,115,118].

While the template DNA loop model has been proposed to function in nucleosome retention during transcription elongation, it has also been suggested that the formation of such an intra-nucleosomal loop may cause RNA polymerase arrest, probably due to the stable nature of the nucleosome with the template DNA loop [13,113,118,119,120]. In particular, a small intra-nucleosomal DNA loop is reportedly formed more efficiently on a template containing a single-strand break in the non-template strand, than on the undamaged DNA template [118]. This finding suggested that RNAP may be arrested by template DNA looping at sites of DNA damage in chromatin [118]. The histone tails may facilitate this RNAP arrest [58]. Since the arrested RNAP functions as an initiation signal for transcription-coupled DNA repair, template DNA looping may contribute to the detection of DNA damage [118,120].

## 10. Structures of the Template DNA Loop during Transcription Elongation

**Figure 7:**
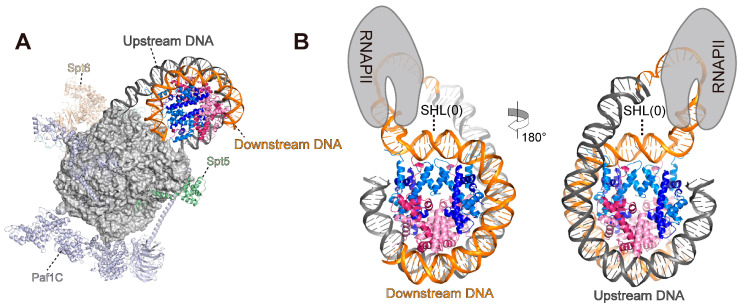
RNAPII–nucleosome structure with template DNA loop. (**A**) The overall structure of the RNAPII elongation complex—nucleosome (PDB: 7UNC). (**B**) Nucleosome structure with template DNA loop, extracted from the overall structure. The downstream and upstream DNAs are colored orange and yellow, respectively.

Various RNA polymerase–nucleosome structures forming the template DNA loop have been described [41,118]. Filipovski et al. reported a cryo-EM structure in a nucleosome–EC containing PAF1C, SPT6, and SPT4/5 (DSIF), in which the leading edge of the RNAPII reaches around the SHL(−0.5) position of the nucleosome (Figure 7A). In this structure, the upstream DNA segment emerging from the RNAPII exit site is once again bound to the exposed surface of the histone octamer in the downstream nucleosome, forming a ~90 bp DNA loop. In the nucleosome, ~55 bp of the upstream DNA are rewrapped on the promoter-proximal side of the nucleosome from SHL(−6.5) to SHL(−1). The path of the rewrapped DNA on the nucleosome is nearly the same as that on the intact nucleosome (Figure 1A and Figure 7B). The promoter-distal side of the nucleosomal DNA around SHL(+7)–(+5) is unwrapped in a similar way to the structure of the RNAPII EC–FACT–nucleosome complex at the SHL(0) position (Figure 5B and Figure 7B), confirming that the unwrapping of distal nucleosomal DNA occurs when RNAPII reaches SHL(0) [42]. Gerasimova et al. reported a low-resolution structure in an *E. coli* RNA polymerase–nucleosome complex, in which the active center of the RNAP reached the 24th base from the nucleosome entry (+24) and backtracked to the +20 position. In this experiment, a single-strand break was inserted into the non-template strand DNA at the +12 position to induce RNAP arrest and template DNA loop formation. In this structure, the upstream DNA behind the RNAP bound to the surface of the histone octamer again, forming a ~55 base-pair template DNA loop that locks RNAP in the arrested state.

These two structures reveal that template DNA loop structures are formed at various RNAP pausing positions with diverse loop sizes, which may decrease the possibility of nucleosome loss [118]. Transcription elongation factors, histone chaperones, and histone modifications may contribute to the recovery of RNAP from the template DNA loop-induced arrested state [13]. The template DNA loop formation when RNAP reaches SHL(0) may be especially important, because the histone complex would be dissociated from the downstream DNA by RNAP passing through SHL(0). In the template looping model, after passing through SHL(0), the histone complex would be transferred upstream of RNAP via the template DNA loop, resulting in nucleosome reassembly and efficient RNAP transcription of the downstream naked DNA.

## 11. Concluding Remarks and Perspectives

A series of dynamic structural transitions of the nucleosome during transcription have been revealed. The structures of FACT-mediated and template DNA looping-mediated nucleosome reassembly during transcription demonstrate that FACT binding to the nucleosome and loop formation both require nucleosome deformation, in which the promoter-proximal H2A–H2B is exposed to the solvent due to the DNA detachment promoted by RNAPII progression [39,41,42,118]. Since FACT and the template DNA both bind to the exposed H2A–H2B, they could be mutually exclusive. Future studies are needed to clarify how these pathways are selected and whether they are compatible. In addition, the promoter-proximal H2A–H2B dimer could be dissociated [40,102]. Since the H2A–H2B dimer appears to be the key to nucleosome retention through the template DNA looping and/or FACT, it will be intriguing to determine whether the nucleosomes are retained during transcription in the absence of the H2A–H2B dimer.

Recently, the structure of the H3–H4 octasome, consisting of two molecules of the H3–H4 tetramer, was reported [121,122]. The H3–H4 octasome wraps approximately 120 base pairs of DNA, forming a nucleosome-like structure, and lacks the acidic patch on its surface due to the absence of the H2A–H2B dimer. In vivo cross-linking experiments suggested that the H3–H4 octasome exists in cells [122]. Since FACT, as well as many nucleosome remodelers, requires the H2A–H2B molecule for nucleosome binding, the absence of H2A–H2B is likely to affect nucleosome occupancy, nucleosome dynamics, and higher-order chromatin folding. Therefore, in the near future, it will be exciting to investigate the mechanism by which RNAPII transcribes and passes through the H3–H4 octasome.

In this review article, we focused on nucleosome dynamics during FACT-mediated and template DNA looping-mediated pathways. In addition to FACT, other histone chaperones, such as HIRA, Spt6, and Nap1, participate in nucleosome transcription [6,92,94,123,124]. Although FACT is plentiful in yeast, undifferentiated cells, and many cancer cells, it is reportedly not as abundant in differentiated cells. Instead, LEDGF and HDGF2 have been proposed to take over the roles of FACT in differentiated cells [125]. How these histone chaperones are selected, and how they reorganize nucleosomes during transcription, are future intriguing questions. In addition, the contribution of template looping to nucleosome reassembly in vivo remains unknown. Deciphering diverse nucleosomes with histone chaperones, modifications, and template DNA looping in various transcription contexts through structural, cell biological, and genome-wide analyses will provide insights toward understanding chromatin transcription and epigenetic information maintenance.

## Figures and Tables

**Table 1:** Features of Spt16 binding modes.

Spt16 Mode		1	2	3
Nucleosome conditions	H2A–H2B presence on Spt16 side	Yes	Yes	Yes
	SHL(−7)–(−4) DNA peeling	Yes	Yes	Yes
	SHL(−7)–(−1) DNA peeling	No	Yes	No
	H2A-docking domain disruption on Spt16 side	No	Yes	No
Binding states	CTD binding to H2A–H2B	Yes	Yes	Yes
	MD binding to H3–H4	No	Yes	No
	Linker helix binding to acidic patch	/	Yes	No
	MD position on nucleosomal DNA	SHL(−0.5)–(+1)	SHL(−0.5)–(+1)	SHL(−1)–(0)
PDB IDs		7NKY	7XSX, 7XTI, 7XTD	6UPK, 6UPL, 7XT7

**Table 2:** Features of Ssrp1/Pob3 binding modes.

Ssrp1/Pob3 Mode		1	2	3
Nucleosome conditions	H2A–H2B presence on Pob3 side	Yes	Yes	No
	SHL(+5)–(+7) DNA peeling	Yes, No	Yes	Yes
	H2A-docking domain disruption on the Ssrp1/Pob3 side	No	No	/
Binding states	MD binding to H3–H4	No	No	Yes
	MD position on nucleosomal DNA	SHL(+1)	SHL(+0.5)	SHL(+0.5)
PDB IDs		7NKY, 7XSX, 7XTD	6UPL, 7XT7	6UPK, 7XTI

**Table 3:** Features of DD binding modes.

DD Mode	1	2
Spt16 mode	1, 2	3
Ssrp1/Pob3 mode	1, 3	2, 3
DD binding to nucleosomal DNA	SHL(+0.5)	SHL(−0.5)
PDB IDs	7NKY, 7XSX, 7XTI, 7XTD	6UPL, 6UPK, 7XT7

**Table 4:** FACT–nucleosome complex structures.

PDB ID	Description	Spt16 MD Mode	Ssrp1/Pob3 MD Mode	DD Mode	Reference
7NKY	EC paused at SHL(−4)	1	1	1	[39]
7XSX	EC paused at SHL(0): EC49	2	1	1	[42]
7XT7	EC paused at SHL(0):EC49B Histone octamer jumped downstream of EC49	3	2	2	[42]
7XTI	EC paused at SHL(+1):EC58hex histone hexamer upstream of the EC	2	3	1	[42]
7XTD	EC paused at SHL(+1):EC58oct Histone octamer upstream of the EC	2	1	1	[42]
6UPL	FACT-octasome complex reconstituted by mixing H2A–H2B dimer, H3–H4 tetramer, and FACT	3	2	2	[60]
6UPK	FACT-hexasome complex reconstituted by mixing H2A–H2B dimer, H3–H4 tetramer, and FACT	3	3	2	[60]

## Data Availability

No new data were created or analyzed in this study. Data sharing is not applicable to this article.
